# The role of the pulmonary function laboratory to assist in disease management: pulmonary hypertension

**DOI:** 10.36416/1806-3756/e20240033

**Published:** 2024-05-08

**Authors:** Eloara V M Ferreira, Juliana S Lucena, Rudolf K F Oliveira

**Affiliations:** 1. Disciplina de Pneumologia, Escola Paulista de Medicina, Universidade Federal de São Paulo - UNIFESP - São Paulo (SP) Brasil.

## INTRODUCTION

Pulmonary arterial hypertension (PAH) is a rare disease associated with high mortality due to right ventricular dysfunction. During PAH diagnostic workup, pulmonary function tests (PFTs) are essential to exclude relevant parenchymal lung disease. PFTs additionally provide information on PAH severity and prognosis.

## OVERVIEW

A 22-year-old woman reported a five-year history of progressive dyspnea, functional class II. After a detailed diagnostic workup, an idiopathic PAH (IPAH) diagnosis was made, and bosentan was started. Baseline PFT revealed an obstructive ventilatory pattern with normal lung volumes and a decreased DL_CO_ (Panel 1A-C). She had never smoked and had no previous asthma diagnosis. During follow-up, the patient was treated with add-on triple therapy and was listed for lung transplantation. Eleven years after the onset of the disease, enlarged pulmonary arteries were evident by chest imaging. PFT showed a decrease in FVC, FEV_1_, and FEV_1_/FVC, with signs of air trapping and reduced DL_CO_ and K_CO_ (Panel 1D-F). In patients with PAH, a decreased DL_CO_ is expected; however, without lung volume abnormalities. Additionally, DL_CO_ is a marker of disease severity,^1^ ^-^ ^3^ and a DL_CO_ < 45% is related to aging, smoking history, lower exercise capacity, and worse survival on PAH.^3^ K_CO_ adds information to PFT interpretation, and low levels associated with normal Va are suggestive of pulmonary vascular disease or intrapulmonary right-to-left shunting, signaling inefficient gas exchange.^4^ Both restrictive and obstructive PFT patterns have been described in PAH patients. It has been demonstrated that the worsening of lung function is related to PAH severity.^1^ ^-^ ^3^ Notably, obstructive disturbance among PAH patients is more frequently found in congenital heart disease and connective tissue disease.^3^ A recent study has demonstrated that patients with IPAH without lung disease have a better five-year-survival compared with those with mild lung disease (70% vs. 22%, respectively; p < 0.0001). However, the mechanisms underlying lung function disturbances in PAH remain unclear. The hypotheses of airflow obstruction are speculative, as an inflammatory response could have similar effects on vascular and airway smooth musculature, causing a proliferation in small airway wall thickening. Another possible explanation includes “competition for space” between hypertrophied vessels and distal airways within the interstitial space.^1^ ^,^ ^2^ Recently, Rahaghi et al. analyzed PFT at baseline and at the time of lung transplantation in PAH and found a reduction in FEV_1_, FVC, and FEV_1_/FVC over time. There was no evidence of parenchymal or airway disorder in the pathology. Airflow obstruction correlated best with an expanded thoracic blood volume and increased pulmonary artery diameter despite unchanged pulmonary hemodynamics. In this context, airway compression secondary to pulmonary arteries dilatation may be a potential mechanism of peripheral airway obstruction in PAH.^4^ Regarding DL_CO_, the reduction could be explained by increased alveolar-capillary membrane thickness related to endothelial cell proliferation and reduced perfused pulmonary capillary bed. However, the loss of alveolar-capillary membrane diffusing capacity and lung capillary blood volume could also explain a reduced DL_CO_ in PAH.^1^ ^,^ ^2^ ^,^ ^5^

## CLINICAL MESSAGE

PFTs provide valuable information to monitor disease severity and prognosis in PAH. There is a continued interest in understanding the pathophysiology of lung function disturbances in PAH, aiming to improve PAH phenotyping and the potential impact on new targeted treatment approaches. An approach for PFT interpretation in the scope of chronic dyspnea and the investigation of PH etiology is described (Panel 1G).

**Panel 1:**
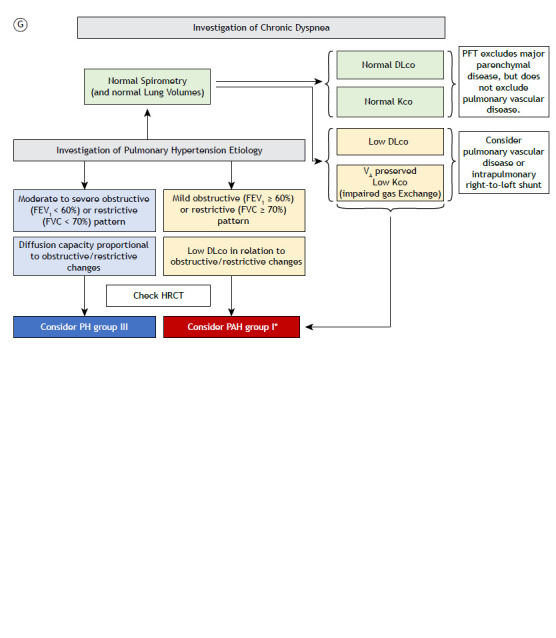
Female, 22 years of age, functional class II at baseline, diagnosed with idiopathic pulmonary arterial hypertension. Echocardiogram showed dilatation of the right chambers and increased systolic pulmonary pressure (sPAP = 50 mmHg), confirmed by right heart catheterization. In A, a chest X-ray was normal (a slight increase of left mediastinum). In B, an electrocardiogram (I, II, and III derivations) revealed right heart chambers overload. In C, baseline pulmonary function test (PFT) revealing mild obstructive ventilatory pattern, normal lung volumes, and a slightly decreased DL_CO_. Follow-up after 11 years, one year before lung transplantation, functional class III, echocardiogram with sPAP = 116 mmHg: in D, a chest X-ray showing increased pulmonary trunk and right interlobar artery; in E, a chest CT scan showing aneurismatic pulmonary trunk and central pulmonary arteries, with centrilobular nodules in parenchyma; and in F, PFT results showing moderate obstructive ventilatory pattern with air trapping and decreased DL_CO_ and K_CO_. In G, a flow chart showing the PFT approach for investigation of chronic dyspnea and pulmonary hypertension: consider interpretation according to normal or altered spirometry (arrows). *Similar findings for patients with chronic thromboembolic pulmonary hypertension without comorbidities. MEF_25%-75%_: mid-expiratory flow; Va: alveolar volume; PH: pulmonary hypertension; and PAH: pulmonary arterial hypertension.
